# Spherical Li_4_Ti_5_O_12_/NiO Composite With Enhanced Capacity and Rate Performance as Anode Material for Lithium-Ion Batteries

**DOI:** 10.3389/fchem.2020.626388

**Published:** 2020-12-15

**Authors:** Jiequn Liu, Shengkui Zhong, Qingrong Chen, Luchao Meng, Qianyi Wang, Zhijian Liao, Jian Zhou

**Affiliations:** ^1^School of Marine Science and Technology, Hainan Tropical Ocean University, Sanya, China; ^2^School of Iron and Steel, Soochow University, Suzhou, China

**Keywords:** lithium-ion batteries, Li_4_Ti_5_O_12_, NiO, composite materials, spray-drying method

## Abstract

Compositing with metal oxides is proved to be an efficient strategy to improve electrochemical performance of anode material Li_4_Ti_5_O_12_ for lithium-ion batteries. Herein, spherical Li_4_Ti_5_O_12_/NiO composite powders have been successfully prepared via a spray drying method. X-ray diffraction and high-resolution transmission electron microscopy results demonstrate that crystal structure of the powders is spinel. Scanning electron microscopy results show that NiO uniformly distributes throughout Li_4_Ti_5_O_12_ matrix. It is found that compositing with NiO increases both discharge platform capacity and rate stability of Li_4_Ti_5_O_12_. The as-prepared Li_4_Ti_5_O_12_/NiO (5%) exhibits a high initial discharge capacity of 381.3 mAh g^−1^ at 0.1 C, and a discharge capacity of 194.7 mAh g^−1^ at an ultrahigh rate of 20 C.

## Introduction

Lithium-ion batteries (LIBs) have powered our daily life from the digital equipment to electric vehicles (EVs) due to their superior energy density compared with conventional rechargeable batteries (Nitta et al., 2015; Li et al., 2018; Sui et al., 2020a,b). Graphite is broadly used as the anode material for LIBs due to broad charge/discharge plateau and low cost. However, previous studies reported that a passive layer can easily form on graphite surface, which leads to decrease in capacity (Ma et al., 2013; Ding et al., 2016; Heng et al., 2020). In addition, the lithium intercalation potential of carbon is close to the lithium metal, which tends to cause growth of lithium dendrite and induce serious safety issues. Thus, numerous studies have been carried out to develop alternative anode materials (Su et al., 2018; Xiao et al., 2018; Zhao et al., 2018; Wu et al., 2019; Zheng et al., 2020).

Li_4_Ti_5_O_12_ (LTO), a well-known intercalation compound, is one of such materials due to its stable cycling performance (Cunha et al., 2019; Natarajan et al., 2019; Nasara et al., 2020). The outstanding stability is ascribed to the “zero-strain” behavior upon Li^+^ insertion/extraction (Goodenough and Park, 2013). LTO, with a Fd-3m space group crystal structure and a lattice parameter 8.3595 Å, can be denoted as [Li]^8a^[Li_1/3_Ti_5/3_]^16d^[O_4_]^32e^ (He et al., 2017; Mandal et al., 2018; Zheng et al., 2018; Wang et al., 2020). In this structure, 75% Li^+^ ions occupy the tetrahedral 8a sites and the remaining Li^+^ ions together with all Ti^4+^ ions occupy the octahedral 16d sites. During intercalation process, LTO undergoes a structure transformation into rock-salt, i.e., when Li^+^ ions in 8a sites combine with external Li^+^ ions, lithium migrates to 16c octahedral sites: [Li]^8a^[Li_1/3_Ti_5/3_]^16d^[O_4_]^32e^ + 3Li^+^ + 3e^−^ → [Li_2_]^16c^[Li_1/3_Ti_5/3_]^16d^[O_4_]^32e^. As a result, the electrochemical response exhibits a flat and wide voltage platform at 1.55 V vs. Li^+^/Li, which is sufficiently high to prevent formation of Li dendrites (Xu et al., 2016; Yao et al., 2018; Zheng et al., 2018).

In spite of high stability, LTO is of moderate rate capability and capacity decay during long-term cycling (Chiu et al., 2016; Yuan et al., 2017). Recently, many researchers devoted to enhance cyclic stability and rate capability of LTO by coating/compositing/doping various materials (Yang and Gao, 2009; Du et al., 2011; Hou et al., 2019; Alia et al., 2020). In particular, transitional metal oxides (Sha et al., 2013; Wang et al., 2013; Chen et al., 2014; Yi et al., 2014) draw much attention because of their high specific capacity and volumetric energy density.

LTO is usually prepared by solid-state synthesis methods using TiO_2_ and Li_2_CO_3_ or LiOH as raw materials (Lin and Duh, 2011; Liu et al., 2015; Wu et al., 2017; Li et al., 2020). However, the products synthesized by these methods suffer from wide size distribution and impurities. Therefore, solution techniques such as sol-gel, hydrothermal synthesis and spray drying have been proposed to synthesize high-purity and nanocrystalline LTO (Xiang et al., 2011; Wen et al., 2015; Wu et al., 2018; Xue et al., 2019; Yang et al., 2019). Among these techniques, spray drying can produce homogeneous chemical composition LTO powders with narrow size distribution at low preparation temperature, which has a significant effect on packing density of the powders. Wen and co-worker (Wen et al., 2015) synthesized spherical LTO with dense nanopores through the spray drying method. The pores are believed to benefit electrolyte transmission and therefore improve charge-discharge performance at the high current rate.

In this work, LTO/NiO composites are prepared via the spray drying method. Effects of NiO content (5, 10, 20 wt.%) on structural properties and electrochemical performance are investigated with various characterization techniques including XRD, SEM, TEM. This work offers an effective strategy to high-performance LTO-based anode materials for LIBs.

## Experimental Section

LTO/NiO powders were synthesized by the following steps. (1) 2.08 g LiOH (A.R.) and *x* g NiO (A.R., *x* = 0.5, 1, 2) powders were dissolved into 600 ml deionized water by continuous stirring to form a green slurry. (2) 37 g C_16_H_36_O_4_Ti (A.R.) was added to 150 ml deionized water and stirred for 10 min to form a white suspension. Afterward, a certain amount of hydrogen peroxide (25 wt.%) was added into this suspension, and then pH value was controlled be 9–10 by adding NH_3_·H_2_O (15 wt.%) dropwise. (3) After stirring for 30 min, yellow-green solution was formed. (4) The green slurry obtained from step (1) was mixed with the yellow-green solution to form a light green transparent solution. The dispersion was then sent through an atomizer using nitrogen as carrier gas. Light green precursor powder was obtained after the transparent solution was passed through the spray dryer. Then, the collected particles were annealed at 650°C under air condition for 4 h, and the final Li_4_Ti_5_O_12_/NiO composites were obtained. Similarly, LTO powders for comparison was obtained by the same method without the addition of NiO.

The crystal structures of the as-obtained powders were characterized by X-ray diffraction (XRD, MERCURY CCDX) with Cu Kα radiation. The particle morphologies of all samples were examined by scanning electron microscopy (SEM, S-4700). High-resolution transmission electron microscopy (HRTEM, FEI TecnaiG220) was used to analyze the microstructure structure.

Working electrode was prepared by mixing 80 wt.% active materials, 10 wt.% carbon black and 10 wt.% PVDF in N-methyl pyrrolidinone to form a slurry, which was then coated onto copper foil. The obtained slurry on current collectors was dried at 120°C for 12 h in vacuum, and the obtained powders were punched to be 14 mm diameter disks. The typical loading amount of active materials in the electrode plate was about 2 mg cm^−2^. Electrochemical performance was tested in a voltage range of 0.01–3 V at 0.1–20 C rates (1 C = 400 mA g^−1^) by a battery test system (Neware, 5 V-20 mA) for CR2025 coin-type cells, which were assembled in an argon-filled glove box. Cyclic voltammograms (CV) were conducted at a scan rate of 0.05 mV s^−1^ between 0.01 and 3 V.

## Results and Discussion

**Figure 1:**
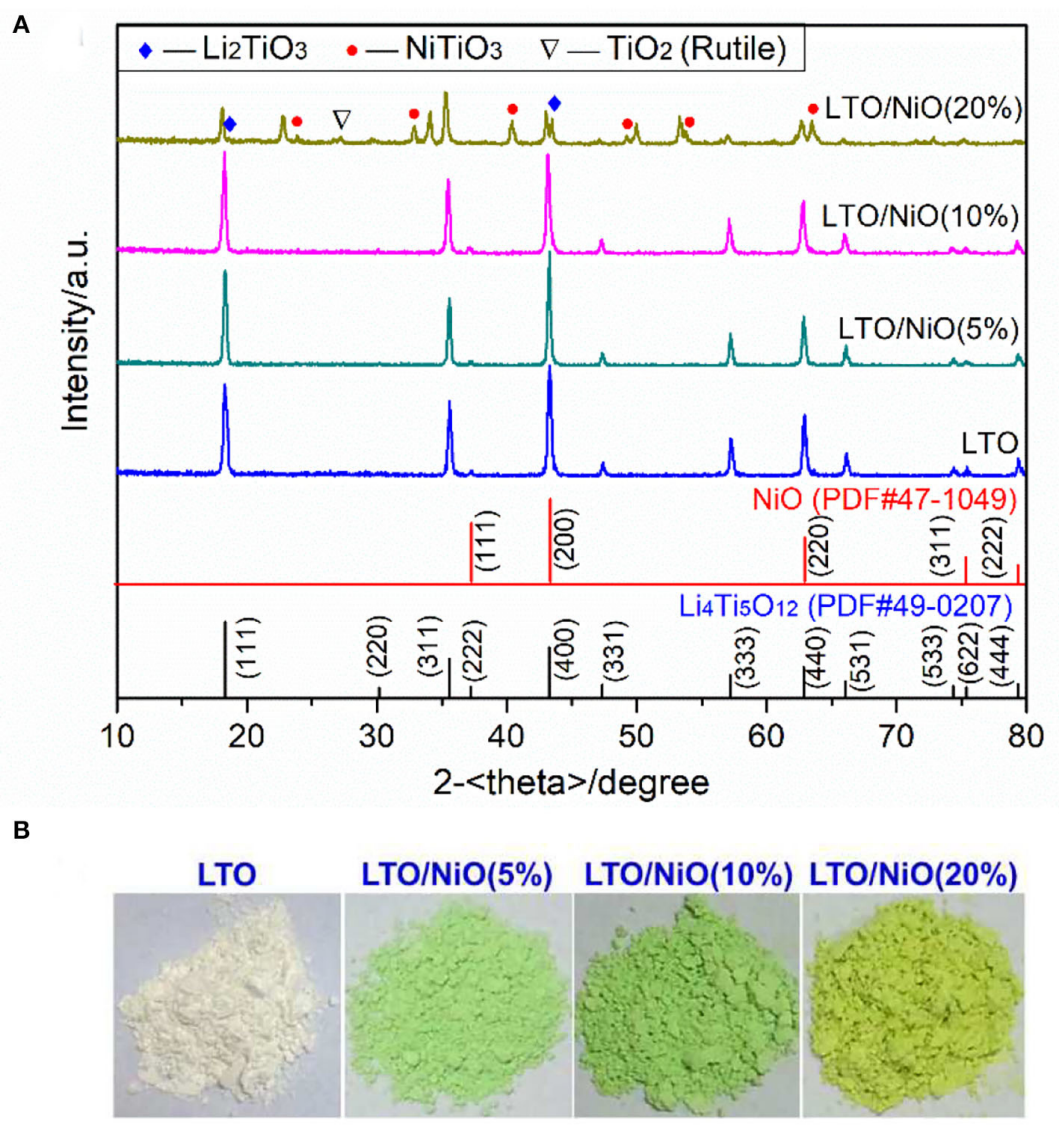
**(A)** XRD patterns of the LTO/NiO samples, **(B)** Images of the LTO/NiO powders.

Crystal structures of the as-prepared powder materials are examined by X-ray diffraction. As shown in Figure 1A, both the LTO (JCPSD No. 49-0207) and the LTO/5-20%NiO (JCPSD No.44-1159) are of the same spinel structure. According to lattice parameter (*a*) summarized in Table 1, addition of NiO from 0 to 20% increases the parameter from 8.3557 to 8.3859 Å, which can be ascribed to that some Ni atoms embedded into the lattice of Li_4_Ti_5_O_12_. Figure 1B shows the appearance of the samples. As we know, the pure NiO powder is green. It can be seen that, as NiO increases, sample color changes, which demonstrates successful synthesis of Li_4_Ti_5_O_12_/NiO composites. It should be noted that there some peaks of impurity phase in the sample of LTO/NiO (20%), which indicates that the 20 wt.% of NiO is too high for the composite.

**Table 1:** Lattice parameter of Li_4_Ti_5_O_12_ phase all the samples.

**Samples**	***a*=*b*=*c* (Å)**	***V* (Å^**3**^)**
LTO	8.3557	583.39
LTO/NiO (5%)	8.3579	583.85
LTO/NiO (10%)	8.3702	586.44
LTO/NiO (20%)	8.3859	589.73

**Figure 2:**
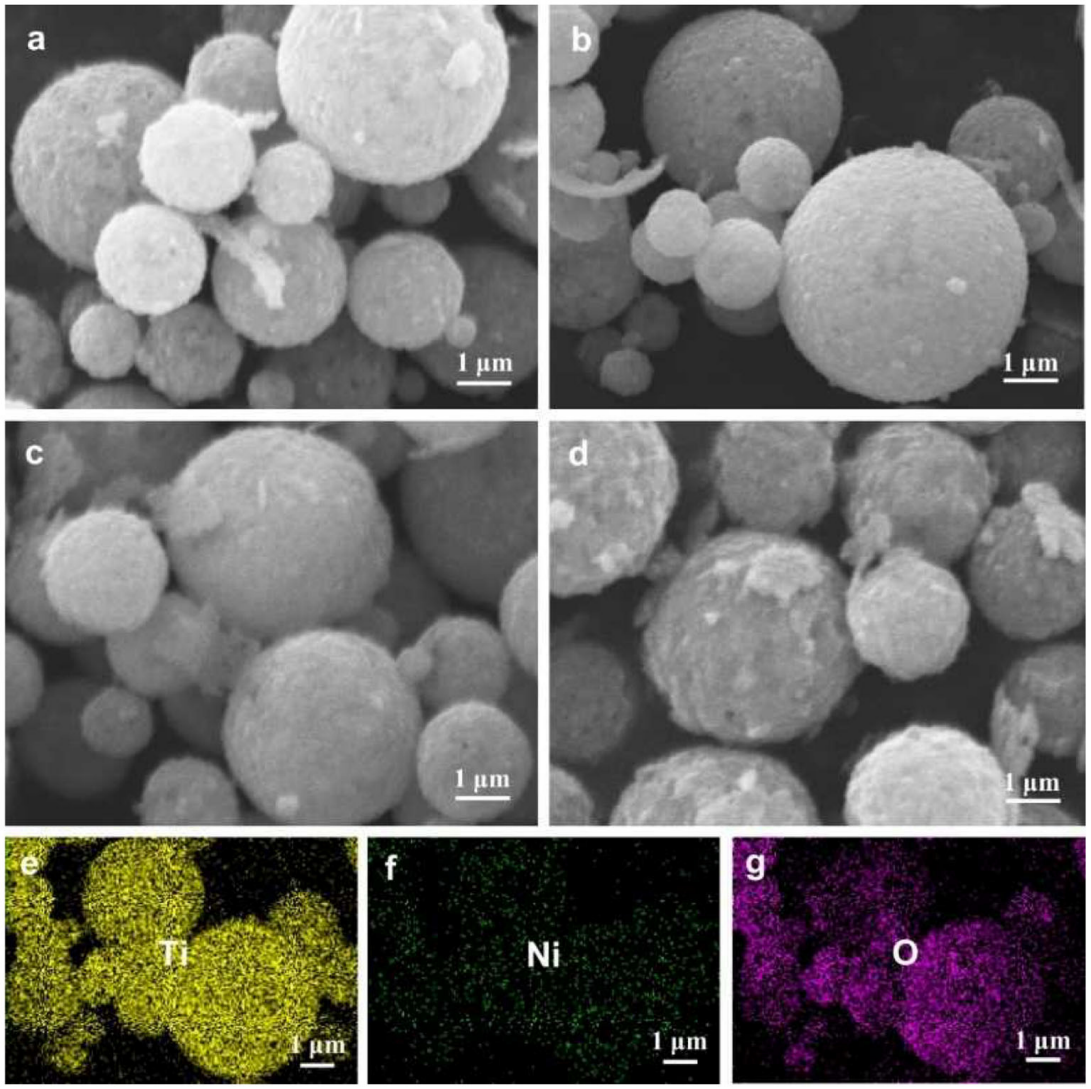
SEM images of **(a)** LTO, **(b)** LTO/NiO (5%), **(c)** LTO/NiO (10%), **(d)** LTO/NiO (20%). EDS elements mapping of **(e)** Ti, **(f)** Ni, **(g)** O for the LTO/NiO (5%).

Typical SEM images of the LTO and the LTO/NiO powders are given in Figure 2. It is found that all the powders are of similar spherical morphology with particle size ranging from about 0.5 to 2.5 μm. In addition, as illustrated in Figures 2e–g, EDS mapping exhibits that Ti, Ni, and O are uniformly distributed, indicating that LTO and NiO were fully mixed.

**Figure 3:**
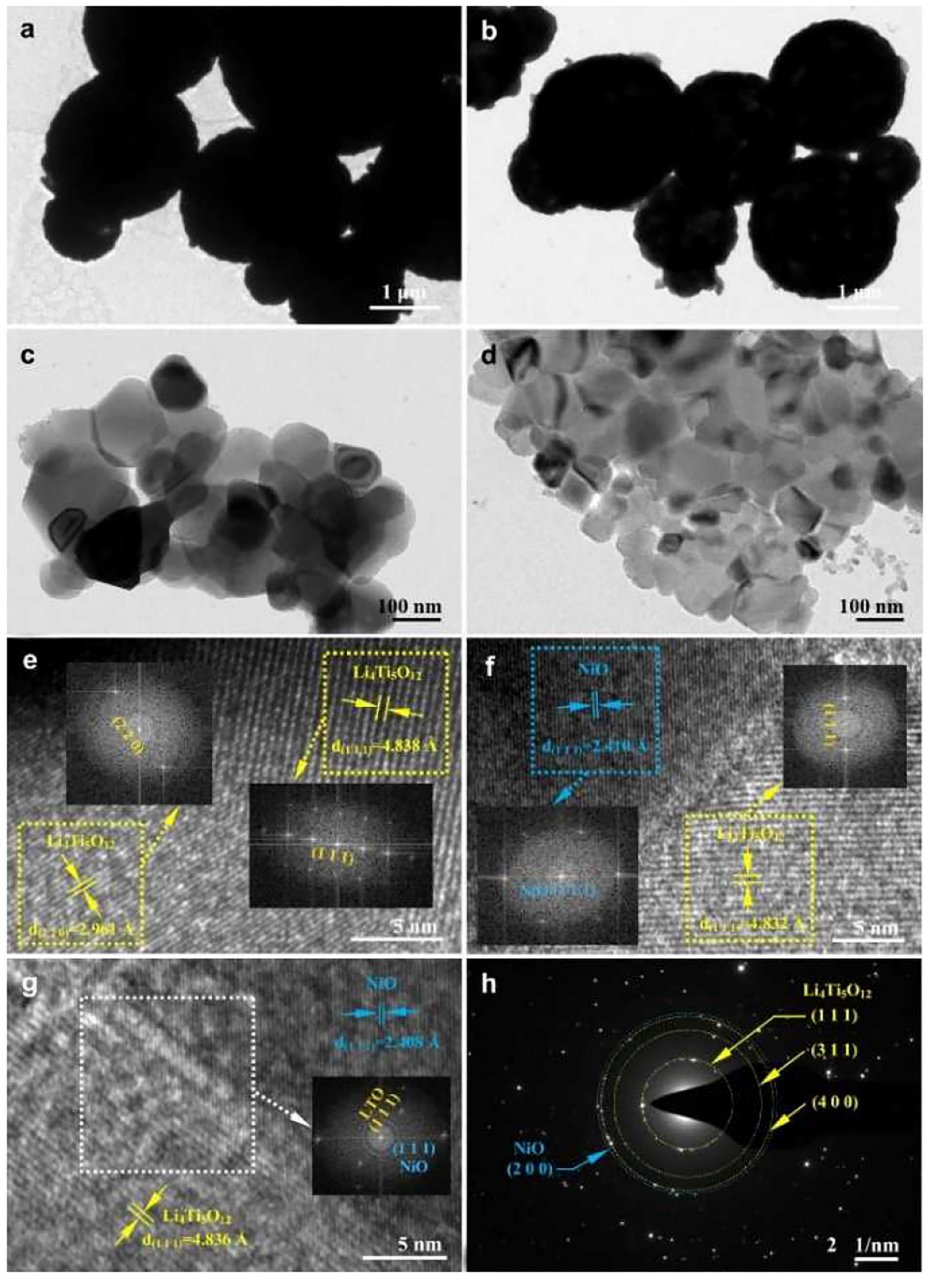
TEM and HRTEM images of **(a,c,e)** LTO and **(b,d,f,g)** LTO/NiO (5%). **(h)** Selected area electron diffraction pattern of the LTO/NiO (5%).

TEM and HRTEM images of the LTO and LTO/NiO (5%) samples are presented in Figure 3. It can be seen from TEM images of both samples (Figures 3a–d) that the average sizes of primary particles are around 200 nm, which is consistent with the SEM results. The primary particles further agglomerate to form secondary particles of a size of about several micrometers. To further characterize microstructure, HRTEM is performed. In Figure 3e, diffraction spots of the corresponding FFT are assigned to (111) plane (*d* = 0.4838 nm) and (220) plane (*d* = 0.4838 nm) of spinel LTO, indicating high-quality crystallinity of LTO. For the LTO/NiO (5%) sample, apart from the spinel LTO with an Fd-3m space group, there are additional (111) diffraction spots emerging in FFT pattern, as seen in Figures 3f,g. Selected area electron diffraction (SAED) pattern in Figure 3h indicates existence of both LTO and NiO phases. Based on all the characterization mentioned above, LTO/NiO composites have been successfully synthesized.

**Figure 4:**
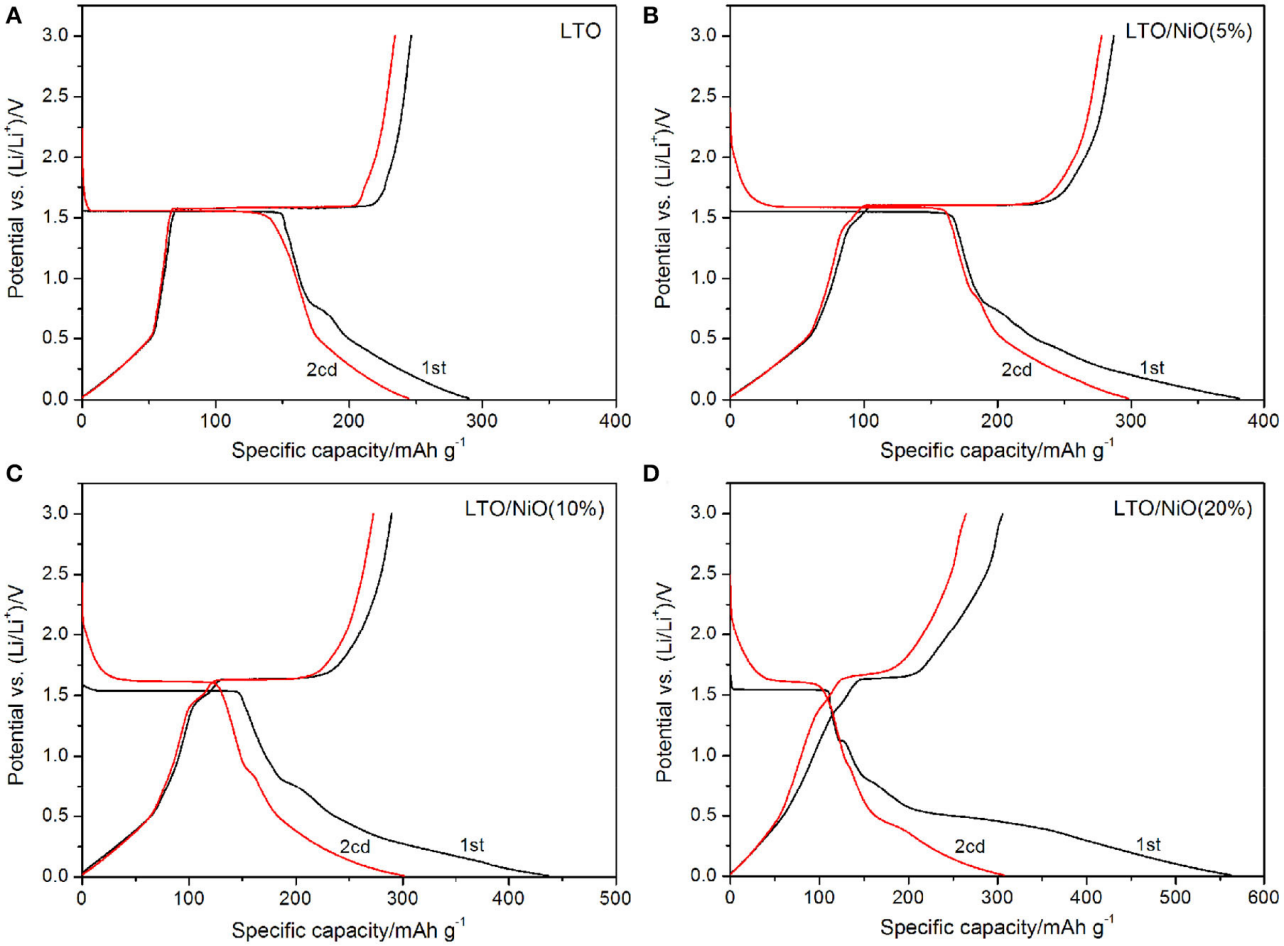
The first two charge/discharge curves at 0.1 C rate of **(A)** LTO, **(B)** LTO/NiO (5%), **(C)** LTO/NiO (10%), **(D)** LTO/NiO (20%).

Electrochemical performances of all the samples are examined in a voltage window of 0.01–3 V. The first two charging-discharging curves are shown in Figure 4. All samples exhibit a voltage platform occurring around 1.55/1.57 V during the initial charging-discharging process (Xu et al., 2016; Yao et al., 2018; Zheng et al., 2018). It is noted that the platform around 1.55/1.57 V becomes shorter and the discharge capacity increases with increase in NiO content. Under the rate of 0.1 C, the pure LTO delivers a discharge capacity of 290.3 mAh g^−1^. In contrast, the discharge capacity increases to 381.3, 436.7, 563.1 mAh g^−1^ for LTO/NiO (5%), LTO/NiO (10%) and LTO/NiO (20%), respectively. At the second charge-discharge cycle, the platform of pure LTO is lower than that of the LTO/NiO composites. Among them, the LTO/NiO (5%) sample exhibits the largest discharge specific capacity of 297.9 mAh g^−1^. In addition, with the increase of NiO amount, the capacity in the low voltage range increases during the first discharge, indicating that NiO can improve the discharge capacity of LTO in the low-voltage section.

**Figure 5:**
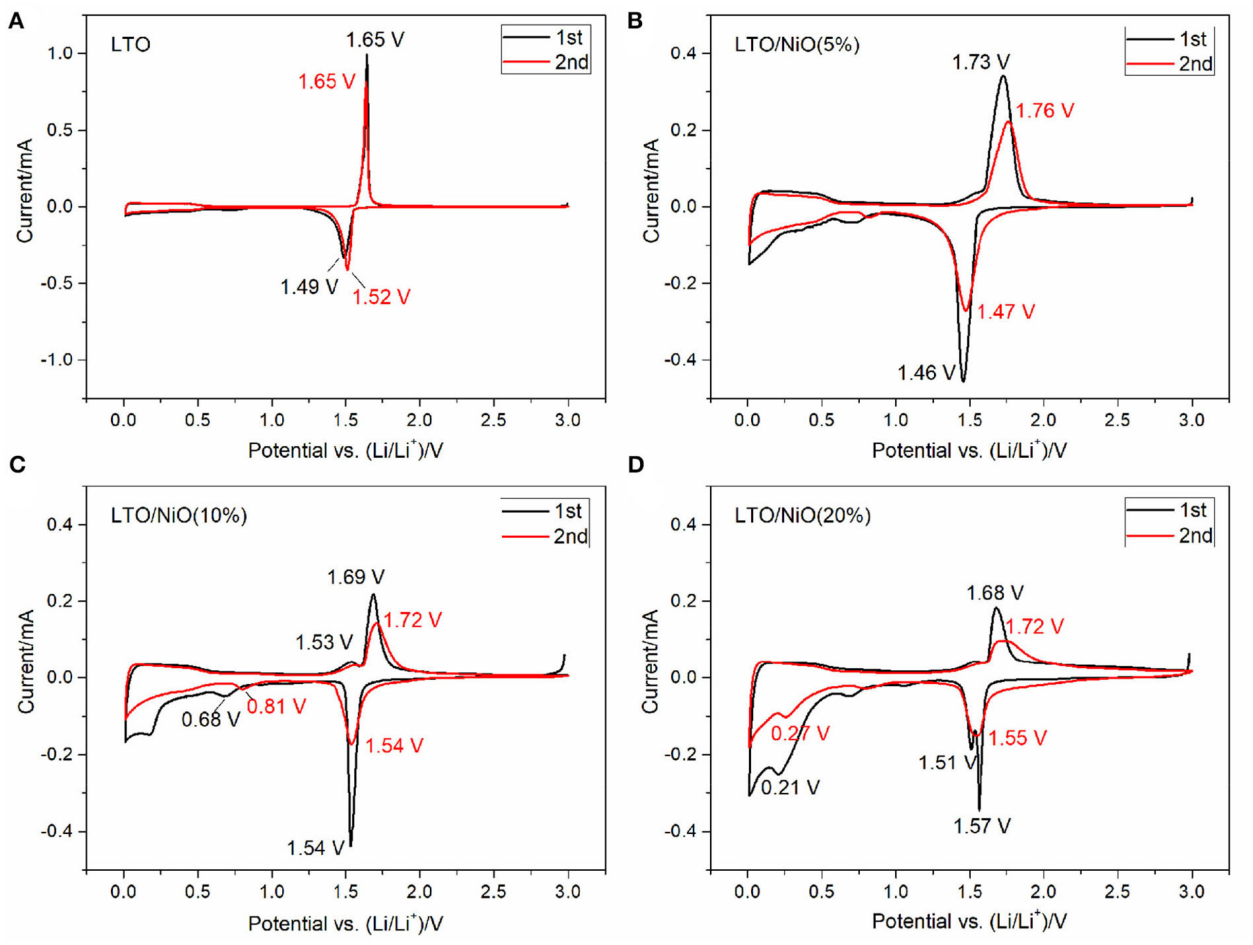
Cyclic voltammetry (CV) curves at a scan rate of 0.05 mV·s^−1^ for **(A)** LTO, **(B)** LTO/NiO (5%), **(C)** LTO/NiO (10%), **(D)** LTO/NiO (20%).

Electrochemical cyclic voltammetry curves of the first two cycles of the LTO and LTO/NiO samples are evaluated at a scan rate of 0.05 mV s^−1^ between 0.01 and 3 V. In Figure 5, an oxidation peak at about 1.5 V and a reduction peak at around 1.7 V appear for all samples, which are ascribed to the oxidation/reduction reactions of the Ti^3+^/Ti^4+^ couple in the cubic structure. It can be found that the patterns of the first and second cycles are almost identical for the LTO sample, which is consistent with the first two charging-discharging curves. In addition, there are additional redox peaks in low voltage range for the LTO/NiO composites, indicating that the composite samples can deliver higher specific capacity in the same voltage range, which can be verified by subsequent electrochemical tests.

**Figure 6:**
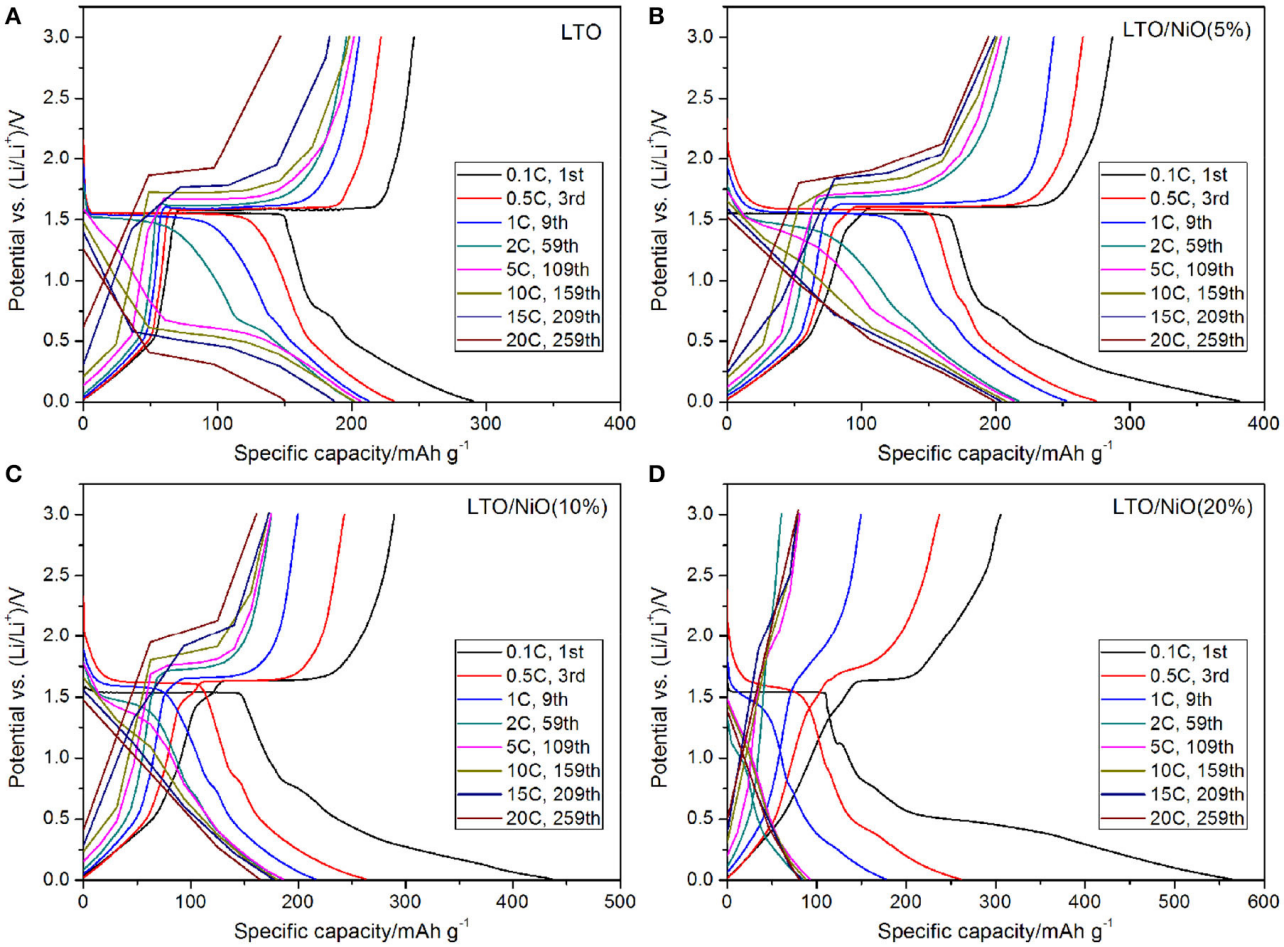
Charge/discharge curves at different rate of **(A)** LTO, **(B)** LTO/NiO (5%), **(C)** LTO/NiO (10%), **(D)** LTO/NiO (20%).

The rate capabilities of the LTO, LTO/NiO (5%), LTO/NiO (10%), and LTO/NiO (20%) electrodes are tested at various C-rates from 1 to 20 C. In Figure 6A, the discharge capacity of pure LTO at 0.1, 1, 2, 5, 10, 15, and 20 C is 290.3, 212.7, 201.2, 206.3, 201.6, 186.6, and 150.1 mAh g^−1^, respectively. Among the LTO/NiO composites (Figures 6B–D), the LTO/NiO (5%) sample shows the best rate capability, exhibiting discharge capacity of 286.9, 243.3, 210.1, 204.2, 200.9, 199.4, and 194.7 mAh g^−1^ at 0.1, 1, 2, 5, 10, 15, and 20 C, respectively. In stark contrast, the LTO/NiO (20%) sample shows the worst rate capability, exhibiting the discharge capacity of 563.1, 260.8, 177.5, 84.3, 92.2, 82.8, and 80.1 mAh g^−1^ at 0.1, 1, 2, 5, 10, 15, and 20 C, respectively. Combined with previous structural analyses, it can be inferred that the degradation of electrochemical performance can be ascribed to the destruction of the original spinel structure of LTO.

**Figure 7:**
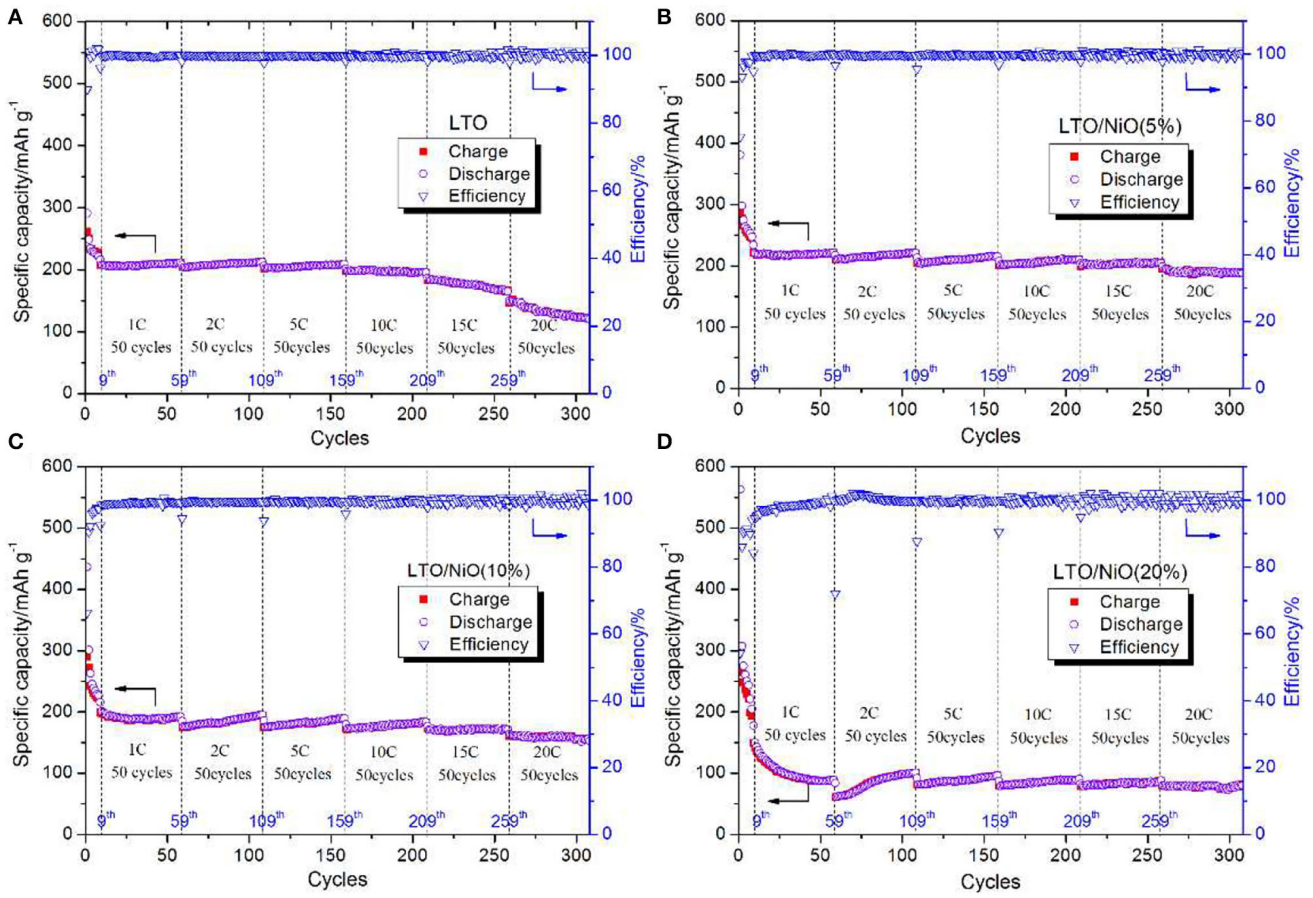
Cycling performance of **(A)** LTO, **(B)** LTO/NiO (5%), **(C)** LTO/NiO (10%), **(D)** LTO/NiO (20%).

Cyclic stability of all samples in a voltage window of 0.01–3 V is tested and shown in Figure 7. As seen, among the four samples, the LTO/NiO (5%) exhibits better electrochemical performance than that of the LTO. After prolong cycles, the LTO/NiO (5%) delivers a specific discharge capacity of 187.6 mAh g^−1^ at 20 C, which is much higher than LTO (120.9 mAh g^−1^). However, when NiO reached 20%, the cycling performance becomes worse, delivering a specific discharge capacity of 80.1 mAh g^−1^ at 20 C. Therefore, appropriate NiO can significantly improve the specific capacity, rate performance and cycling performance of LTO.

## Conclusions

In summary, spherical LTO/NiO composites are successfully prepared by spray drying method. It demonstrates that compositing with NiO is an effective strategy to improve electrochemical performance of LTO. NiO not only increases the discharge platform capacity, but also enhances the rate stability of LTO. As a result, as-prepared LTO/NiO (5%) exhibits a high initial discharge capacity of 381.3 mAh g^−1^ at 0.1 C, and a discharge capacity of 194.7 mAh g^−1^ at an ultrahigh rate of 20 C. This work demonstrates that compositing with transitional metal oxides could enhance the capacity and rate performance of LTO, which is also applied to other anode materials.

## Data Availability Statement

The original contributions presented in the study are included in the article/supplementary material, further inquiries can be directed to the corresponding author/s.

## Author Contributions

JL, SZ, and JZ did the main experiment and wrote the manuscript. QC, LM, QW, and ZL evolved the discussion of the experiment and revised the manuscript. SZ provided the financial support. All authors contributed to the article and approved the submitted version.

## Conflict of Interest

The authors declare that the research was conducted in the absence of any commercial or financial relationships that could be construed as a potential conflict of interest.
